# The Probiotic *Butyricicoccus pullicaecorum* Reduces Feed Conversion and Protects from Potentially Harmful Intestinal Microorganisms and Necrotic Enteritis in Broilers

**DOI:** 10.3389/fmicb.2016.01416

**Published:** 2016-09-21

**Authors:** Venessa Eeckhaut, Jun Wang, Alexander Van Parys, Freddy Haesebrouck, Marie Joossens, Gwen Falony, Jeroen Raes, Richard Ducatelle, Filip Van Immerseel

**Affiliations:** ^1^Department of Pathology, Bacteriology and Avian Diseases, Faculty of Veterinary Medicine, Ghent UniversityMerelbeke, Belgium; ^2^Department of Microbiology and Immunology, Rega Institute for Medical Research, KU LeuvenLeuven, Belgium; ^3^Center for the Biology of Disease, Vlaams Instituut voor BiotechnologieLeuven, Belgium; ^4^Microbiology Unit, Faculty of Sciences and Bioengineering Sciences, Vrije Universiteit BrusselBrussels, Belgium

**Keywords:** *Butyricicoccus*, butyrate, broiler, microbiota, FCR

## Abstract

Probiotics which do not result in the development and spread of microbial resistance are among the candidate replacements for antibiotics previously used as growth promotors. In this study the effect of in-feed supplementation of the butyrate producing *Butyricicoccus pullicaecorum* strain 25-3^T^ on performance, intestinal microbiota and prevention of necrotic enteritis (NE), a disease caused by *Clostridium perfringens* was evaluated in broilers. For the performance study, day old Ross 308 chicks were randomly allocated into two treatment groups and fed either a non-supplemented diet or a diet supplemented with 10^9^ cfu lyophilized *B. pullicaecorum* per kg feed for 40 days. On day 40 broilers administered *B. pullicaecorum* had a significant lower bodyweight (2675 g vs. 2762 g; *p* = 0.0025) but supplementation of *B. pullicaecorum* decreased the feed conversion ratio significantly (1.518 vs. 1.632; *p* < 0.0001). Additionally, ingestion of the *Butyricicoccus* strain significantly lowered the abundance of *Campylobacter* spp. in the caecum and *Enterococcus* and *Escherichia*/*Shigella* spp. in the ileum at day 40. In feed supplementation of *B. pullicaecorum* in the NE trials resulted in a significant decrease in the number of birds with necrotic lesions compared with the untreated control group. These studies show that supplementation of *B. pullicaecorum* is able to improve feed conversion, to reduce the abundance of some potentially important pathogens in the caeca and ileum and to contribute to the prevention of NE in broilers, making the strain a potential valuable probiotic.

## Introduction

Since the 1970’s, broilers have substantially improved in growth rate, breast-meat yield and efficiency of feed conversion (Dawkins and Layton, 2012). Feed conversion ratio (FCR), calculated as the ratio of feed consumed to weight gained, is a widely used performance measure, representing how efficiently the feed is utilized and converted into body mass (Stanley et al., 2012). The extraction of energy and nutrients from feed requires interplay between the biochemical functions provided by the chicken and the intestinal microbiota (Stanley et al., 2014a). The chicken gut harbors a very diverse microbiota that for many years has been kept stable by the use of sub-therapeutic dosages of antimicrobial growth promoters (AGPs) in the feed. The ban on the use of AGPs in the EU has resulted in an increase of intestinal health problems in broiler flocks and thus reduced profitability for poultry farmers. A disease that has emerged after the AGP ban is necrotic enteritis (NE), caused by *Clostridium perfringens*.

Prebiotics, probiotics, essential oils, plant extracts, and organic acids have been proposed as potential alternatives to antibiotics (Alloui et al., 2013). Probiotics or direct-fed microbials (DFM) are live microorganisms that, when administered in adequate amounts, confer a health benefit on the host (Hill et al., 2014). This beneficial effect can be achieved indirectly by improving the properties of the indigenous microbiota, resulting in the protection from enteric pathogen infections (Fuller, 1989). Most probiotic strains that are used in livestock are members of the bacterial genera *Bacillus*, *Enterococcus*, *Lactobacillus* or the yeast genus *Saccharomyces.* They are predominantly used to improve performance parameters, including market-aged body weight and FCR (Gaggia et al., 2010). Probiotics display multiple modes of action, including modification of gut pH via secretion of short chain fatty acids (SCFA), inhibition of the proliferation and colonization of pathogens and control of enteric diseases (van Der Wielen et al., 2000). In feed supplementation of the SCFA butyrate, which is an important end-product of anaerobic bacterial fermentation of carbohydrates, has been shown to decrease pro-inflammatory cytokine expression in broiler challenge models and to induce expression of antimicrobial host defense components in the gut (Sunkara et al., 2011; Zhang et al., 2011). Moreover, it stimulates proliferation, differentiation and function of mucosal epithelial cells and immune cells. Butyrate mediated beneficial effects on growth and intestinal integrity have been demonstrated in piglets (Kotunia et al., 2004; Le Gall et al., 2009) and broiler chickens (Adil et al., 2010; Mountzouris et al., 2010; Ahmed et al., 2014; Ritzi et al., 2014). In all these studies, butyric acid was added to the feed (Guilloteau et al., 2010). A valuable alternative strategy would consist of stimulation of *in situ* production of butyrate through administration of a selected bacterium or substrates that are converted to butyrate.

The objectives of the present study were to assess the effect of dietary supplementation of *Butyricicoccus pullicaecorum*, a butyrate-producing chicken isolate, on animal performance, on the intestinal microbiota composition and on the resistance against *C. perfringens* induced NE.

## Materials and Methods

### Strains

*Butyricicoccus pullicaecorum* strain 25-3^T^ (LMG 24109), isolated from the caecal content of a chicken (Eeckhaut et al., 2008), member of *Clostridium* cluster IV and shown to produce 15.3 mM butyrate under lab conditions, was grown overnight at 37°C in M2GSC medium at pH6 (Miyazaki et al., 1997) in an anaerobic (84% N_2_, 8% CO_2_, and 8% H_2_) workstation (Ruskinn Technology, Bridgend, UK). After centrifugation (6400 × *g*, 37°C, 15 min) of the bacterial culture, the pellet was resuspended and lyophilized in lyoprotectant consisting of 750 ml horse serum and 250 ml distilled water supplemented with 7.5% sucrose (Sigma–Aldrich, St Louis, MO, USA), 0.63% nutrient broth (Sigma–Aldrich, St Louis, MO, USA) and 1 mg/ml cysteine HCl. The number of surviving cells after freeze drying was counted using the agar plate count method and expressed as cfu/g lyophilized powder.

*Clostridium perfringens* strain 56, originally isolated from the intestine of a chicken with severe necrotic gut lesions, was used to induce NE in the *in vivo* broiler model. This strain has been characterized as a NetB toxin positive type A strain (no β2 or enterotoxin genes) and a producer of moderate amounts of alpha toxin *in vitro* (Gholamiandekhordi et al., 2006). Before inoculation of the chickens, the bacteria were cultured in brain heart infusion broth (Oxoid, Basingstoke, UK) for 24 h at 37°C in the anaerobic cabinet.

### Performance Trial

A total of 504 1-day-old (252 males and 252 females) Ross 308 broiler chicks obtained from a commercial hatchery were weighed and randomly allocated to one of two treatment groups. Each treatment group consisted of three replicates with 42 male and three replicates with 42 female chickens per pen. There was no significant difference in body weight between the two groups at the start of the trial. The birds were raised on concrete floor covered with wood shavings. Temperature was maintained at 33°C for the 1st week and then gradually reduced at a rate of 3°C per week to a final temperature of 22°C. The lighting schedule provided 18 h of light per day and proper ventilation was ensured by means of exhaust fans. The animals had *ad libitum* access to water and feed in a mash form that was formulated for a starter (days 1–13), grower (days 14–26), and finisher (days 27–40) period. Lyophilized probiotic bacteria were mixed thoroughly in a small amount of feed (1 kg). The resultant mixture was then mixed with the rest of the feed in a mechanical blender until a thorough and consistent mixture was obtained containing 10^9^ cfu *B. pullicaecorum*/kg feed. All birds were given the same basal diet. The treatment was as follows: basal diet without addition (control) and basal diet containing 10^9^ cfu *B. pullicaecorum*/kg feed.

#### Growth Performance, Feed Conversion Ratio, and Sample Collection

All chickens were weighed individually on d 14, 26, and 40. In addition, feed consumption for each pen between weighing was determined by measuring leftovers of the feed on the same days as the birds were weighed. FCR was calculated.

On days 26 and 40, three chickens per pen were randomly selected and euthanized by an intravenous overdose of sodium pentobarbital 20% (Kela, Hoogstraten, Belgium). The ileal and cecal content was collected yielding 18 digesta samples per treatment and time point. The samples were preserved at -80°C until 100 mg was weighed and subjected to total DNA extraction using hexadecyltrimethylammonium bromide (CTAB) based on the protocol described by Boon et al. (2003). Isolated DNA was used as template for further analyses.

#### Library Construction and Sequencing of the V4 Region of 16S Ribosomal DNA

The V4 region of the bacterial 16S rRNA gene was amplified using PCR primers 515F (5′-GTGCCAGCMGCCGCGGTAA-3′) and 806R (5′-GGACTACHVGGGTWTCTAAT-3′) together with flanking molecular identifier (MID) and Illumina sequencing adapter as described by Kozich et al. (2013). PCR amplification was performed in a 50 μl reaction volume containing 25 μl 2X Taq Master Mix (Thermo Scientific, Aalst, Belgium), 0.2 μM of each forward and reverse primer and 20 ng DNA template. The reaction conditions consisted of an initial 94°C for 3 min, followed by 35 cycles at 94°C for 45 s, 50°C for 60 s and 72°C for 90 s, as well as a final extension at 72°C for 10 min. Next, amplified products with an expected band size of 300–350 bp were checked by 2% agarose gel electrophoresis and ethidium bromide staining. Amplicons were purified using the MoBio UltraClean PCR Clean-Up kit (Mo Bio, Carlsbad, CA, USA) according to the manufacturer’s instructions. Libraries purified from agarose gel electrophoresis were applied for sequencing using Illumina MiSeq sequencer and MiSeq v2 sequencing kit to produce 250 bp pair-end reads. The raw fastq sequences were merged using FLASH software (Magoc and Salzberg, 2011) and quality-filtered using the FASTX-toolkit^1^ Chimeras were removed using UCHIME v6.0.307 (Edgar et al., 2011) and each sample was downsampled to 10,000 reads using random selection of reads. The taxonomy of the reads was determined using RDP classifier (Wang et al., 2007), and species-level OTUs (taxonomical bins of sequences with >97% similarities) were clustered *de novo* using USEARCH v6.0 (Edgar, 2010). All taxonomy tables were created using Perl scripts for the follow-up analysis. Statistical analysis of alpha-diversity and beta-diversity were carried out using Vegan package (Dixon, 2003) in R.

#### Real-Time PCR Quantification of *Campylobacter* spp.

Members of the genus *Campylobacter* were quantified using the CFX384 Bio-Rad detection system (Bio-Rad, Nazareth-Eke, Belgium). Amplification and detection were carried out in a 384-well plate using 2X SensiMix^TM^ SYBR No-ROX mix (Bioline, Nazareth-Eke, Belgium). Each reaction was done in triplicate in a 12 μl total reaction mixture using 2 μl of appropriate dilutions of the DNA sample and 0.5 μM final qPCR primer concentration (**Table 1**). The reaction conditions used were 1 cycle at 95°C for 10 min, followed by 40 cycles at 90°C for 30 s and 60°C for 45 s. A stepwise increase of the temperature from 65 to 95°C was added and melting curve data were analyzed to confirm specificity of the reaction. For construction of the standard curve, a 812 bp long PCR product was generated using standard PCR primers listed in **Table 1** and genomic DNA from *Campylobacter jejuni.* After purification (Invitek, Berlin, Germany) and determination of the DNA concentration with a Nanodrop ND 1000 spectrophotometer (NanoDrop Technologies, Wilmington, DE, USA), the volume of the linear dsDNA standard was adjusted to 10^8^ copies/μl assuming an average molecular weight of 660 per nucleotide pair. This stock solution was serially diluted to obtain a standard series from 10^8^ to 1 copies/μl with each step differing by 10-fold. The copy numbers of samples were determined by reading off the standard series with the cycle threshold (*C*_t_) values of the samples. Gene copy numbers were expressed as log_10_ values per gram wet weight of intestinal content.

**Table 1:** Sequence of used primers.

Target	Forward primer	Reverse primer	Analysis	Reference
16S rRNA gene from *Campylobacter* spp.	CATTGTAGCACGTGTGTC	GGATGACACTTTTCGGAGC	Standard PCR	This study
	nt^∗^ 380–398	nt 1191–1174		
	GCGTAGGCGGATTATCAAGT	CGGATTTTACCCCTACACCA	qPCR	Bui et al., 2012
	nt 526–545	nt 628–647		


*^∗^nt = nucleotide sequences are presented from 5′ to 3′ and their position in the 16S rRNA gene is given based on the sequences of *Campylobacter jejuni* NCTC 11351.*

### Necrotic Enteritis Trials

Ross 308 broiler chickens were obtained as 1-day-old chicks from a commercial hatchery and reared in cages with a density of 30 birds/m^2^ on wood shavings. All cages were separated by solid walls to prevent contact between birds from different treatment groups. Four trials were performed. For each experiment the chickens were randomly allocated into groups of 30 birds. A 23 h/1h light/darkness program was applied. The chickens received drinking water and feed *ad libitum*. The feed was based on wheat/rye as described by Gholamiandehkordi et al. (2007) with soybean meal as protein source. A one and twofold dose of Poulvac Bursa Plus (Zoetis, Zaventem, Belgium) was given in the drinking water on days 4 and 9, respectively, to all groups. On day 11, all birds were orally inoculated with a 10-fold dose of Hipracox (Hipra, Girona, Spain) and on day 16 they received a 10-fold dose of Paracox-8^TM^ (Schering-Plough Animal Health, Brussels, Belgium). Both vaccines were diluted in water and each chicken received 1ml orally. From day 17 onwards, the soybean in the feed was replaced by fishmeal (30%) as the protein source. All chickens were orally challenged (three times a day) on days 18, 19, 20, and 21 with 1 ml of a fresh broth culture containing 5 × 10^8^ cfu *C. perfringens* per milliliter. On day 22 all birds were euthanized by an intravenous overdose of sodium pentobarbital 20% (Kela, Hoogstraten, Belgium). Necrotic lesions in the small intestine (duodenum to ileum) were scored as described by Keyburn et al. (2006). Briefly, the scoring was as follows: 0 = no gross lesions; 1 = congested intestinal mucosa; 2 = small focal necrosis or ulceration (1–5 foci); 3 = focal necrosis or ulceration (6–15 foci); 4 = focal necrosis or ulceration (16 or more foci); 5 = patches of necrosis 2–3 cm long; 6 = diffuse necrosis typical of field cases. Birds with lesions score of two or more were classified as NE-positives.

In the first trial, from day 1 onwards, lyophilized *B. pullicaecorum* bacteria were mixed in the feed of two groups at a concentration of 10^9^ cfu per kg, while the control group received unsupplemented feed. The second, third, and fourth trial both comprised two groups with one group receiving feed supplemented with 10^9^ cfu lyophilized *B. pullicaecorum* per kg and one control group. All the bird experiments were carried out according to the recommendations of, and following approval by the Ethical Committee of the Faculty of Veterinary Medicine, Ghent University (EC2009/098, EC2010/099, EC2012/101, EC2014/50).

### Statistical Analysis

The data for body weight and FCR were statistically analyzed with GraphPad Prism version 5.0 (GraphPad, La Jolla, CA, USA) using a non-parametric unpaired *t*-test. A one-tailed Wilcoxon test for each genus was applied to test for differences in relative abundancy. A non-parametric Mann–Whitney *U* test was performed to compare the mean number *Campylobacter* bacteria as measured with qPCR in the treated and untreated group. The data from the NE trials were analyzed using one-tailed Wilcoxon signed-rank tests. In all cases, a probability level of *p* ≤ 0.05 was considered statistically significant.

## Results

### Performance Trial

#### Daily Weight Gain, Growth Performance, and FCR

The effects of dietary supplementation with *B. pullicaecorum* on animal performance of all broilers and for both sexes separately during different periods are shown in **Table 2.**

**Table 2:** The effect of *Butyricicoccus pullicaecorum* supplementation on the growth performance of the chickens.

		Body weight (g)^a^			Feed conversion ratio^a^		
			
		D14	D26	D40	D0-14	D0-26	D0-40
Male	Ctrl	497.1 ± 4.5	1405 ± 14.3	2949 ± 31.8	1.125 ± 0.013	1.422 ± 0.059	1.629 ± 0.024
	Bp	484.5 ± 4.8	1328 ± 11.2	2789 ± 23.6	1.103 ± 0.006	1.310 ± 0.046	1.521 ± 0.011
	*P*-value	0.0573	**<0.0001**	**<0.0001**	0.1935	0.2043	**0.0148**
Female	Ctrl	466.1 ± 4.7	1250 ± 10.0	2575 ± 23.8	1.111 ± 0.011	1.384 ± 0.008	1.635 ± 0.017
	Bp	463.0 ± 3.9	1268 ± 9.3	2564 ± 18.6	1.100 ± 0.004	1.295 ± 0.002	1.516 ± 0.001
	*P*-value	0.6107	0.1980	0.7061	0.4129	**0.0005**	**0.0120**
All	Ctrl	481.6 ± 3.4	1328 ± 10.2	2762 ± 23.8	1.118 ± 0.008	1.403 ± 0.028	1.632 ± 0.013
	Bp	473.6 ± 3.2	1298 ± 7.5	2675 ± 16.7	1.102 ± 0.003	1.302 ± 0.021	1.518 ± 0.011
	*P*-value	0.0860	**0.0155**	**0.0025**	0.0936	**0.0157**	**<0.0001**


*Body weight and feed conversion ratio (FCR) were measured at three time intervals. ^*a*^Values are the mean of three replicates of 42 chickens ± standard error of the mean. Ctrl: feed without probiotic supplementation, Bp: feed supplemented with 10^*9*^ cfu *B. pullicaecorum* per kg feed. Bolded values are the ones that are significantly different.*

The male birds fed the *B. pullicaecorum* supplemented diet had a lower (*p* < 0.05) body weight on days 26 and 40 than the male birds in the control group. Supplementation of the probiotic had no significant effect on the body weight of the females at the three different time points. Significant differences between treatments were noted in the FCR. The FCR for *B. pullicaecorum* treated females was significantly lower during grower (1.295 ± 0.002 vs. 1.384 ± 0.008) and finisher (1.516 ± 0.001 vs. 1.635 ± 0.017) period than for the control group. A significantly lower FCR for the *B. pullicaecorum* treated male birds (1.521 ± 0.011 vs. 1.629 ± 0.024) was observed only during the finisher period.

#### Characterization of Microbial Communities

**FIGURE 1:**
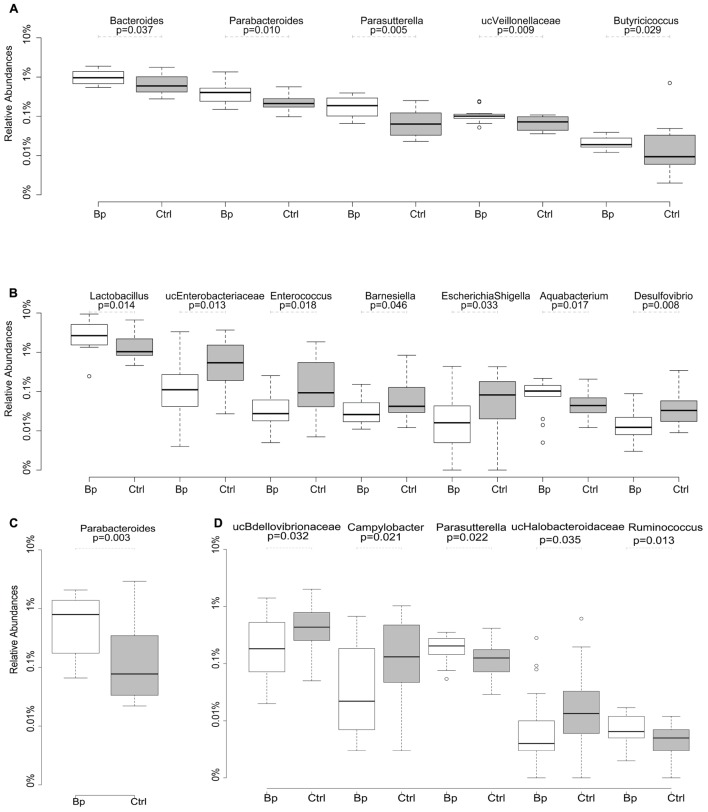
**Relative abundance (percent) of major bacterial genera determined using Ilumina MiSeq sequencing, that showed significant differences in ileum at day 26 **(A)** and day 40 **(B)**, and in caecum at day 26 **(C)** and day 40 **(D)** between the ctrl (received unsupplemented feed) and the Bp (received feed supplemented with 10^9^ cfu *Butyricicoccus pullicaecorum* per kg feed) group.** Abundances are expressed in log scale. o, outliers.

After quality trimming, a total of 745,182 and 980,262 sequencing reads were obtained from the ileal and caecal samples, respectively. Rarefaction of all samples to 10,000 reads resulted in the identification of a total of 7490 and 4996 OTUs in the ileum and caecum, respectively. *Firmicutes*, *Bacteroidetes*, and *Proteobacteria* were the most abundant bacterial phyla in the ileal and caecal samples, regardless age or group. Overall, major separation was observed between the microbiome from different sampling sites (ileum vs. caecum, 21.5% variation, *p* = 0.001); minor differences were noted between samples taken at different time points (26 days vs. 40 days, 6.9% variation, *p* = 0.001). Neither gender nor administration of *B. pullicaecorum* had a significant impact on microbial community differences (both <1% variation, *p* > 0.05). On both days 26 and 40 the proportions of a number of genera representing at least 1% of sequences were altered significantly in the *Butyricicoccus* treated group compared with the control group. On day 26, significantly higher proportions of *Bacteroides*, *Butyricicoccus*, *Parabacteroides*, *Parasutterella*, and unclassified *Veillonellaceae* were detected in the ileum of the probiotic group as compared to control chickens (**Figure 1A**). The numbers of *Lactobacillus* and *Aquabacterium* spp. were significantly higher in the ileum of the treated chickens on day 40, while the abundances of *Enterococcus*, *Escherichia/Shigella*, *Desulfovibrio*, *Barnesiella*, and unclassified *Enterobacteriaceae* were significantly lower in the ileum compared to the control animals (**Figure 1B**). Differences in the caecum on day 26 were observed for *Parabacteroides* (**Figure 1C**) and on day 40 for *Parasutterella* and *Ruminococcus*, with each genus being significantly more abundant in the animals that received *Butyricicoccus*. Numbers of unclassified *Bdellovibrionaceae*, unclassified *Halobacteroidaceae*, and *Campylobacter* were significantly lower in treated vs. control chickens (**Figure 1D**).

#### Quantification of *Campylobacter* spp.

**FIGURE 2:**
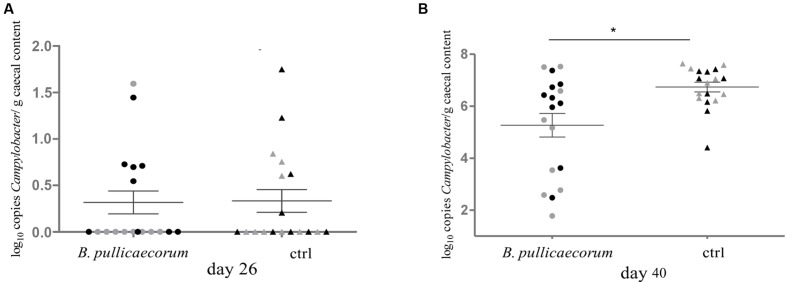
**Quantification using qPCR of *Campylobacter* bacteria in caecal content from nine male (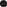) and nine female (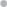) *B. pullicaecorum* treated and nine male (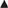) and nine female (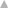) untreated chickens on day 26 **(A)** and day 40 **(B)**.** Bacterial density is expressed as log_10_ copy number of 16S rRNA genes of *Campylobacter* per g of caecal content. ^∗^*p* = 0.0169.

To confirm the decrease in abundance of *Campylobacter* in the caeca of chickens fed a *B. pullicaecorum* supplemented feed, as measured by pyrosequencing, we quantified the number of this human enteropathogen in the chicken intestinal microbiota by qPCR. We found that the average number of bacteria belonging to the genus *Campylobacter* on day 40 was significantly (*p* = 0.0169) lower (5.27 ± 0.46 vs. 6.74 ± 0.19 in log number of 16S rRNA gene copy number/g caecal content) in the caeca of chickens that were administered *B. pullicaecorum* supplemented feed compared to control animals (**Figure 2**).

### Necrotic Enteritis Trials

**Table 3** shows the percentage of birds with necrotic lesions in their small intestine in all trials. The NE challenge was effective in inducing gross lesions, presented as multiple necrotic foci in the duodenum and jejunum. No marked clinical signs or mortality were seen during the experiments. The percentage of birds presenting lesions in the infected untreated control group varied between 14.8 and 56.6. When the results of all trials were combined and evaluated for statistical differences, supplementation of *B. pullicaecorum* resulted in a significant (*p* = 0.0313) reduction in the number of birds developing necrotic lesions in comparison with the untreated control group.

**Table 3:** Percentage of chickens with macroscopic necrotic enteritis (NE) lesions (lesion score ≥2) per trial after challenge with *Clostridium perfringens*.

	Percentage animals with necrotic lesions	
	
Trial	Ctrl	*B. pullicaecorum*
1	56.6	40.0 and 39.3
2	36.0	7.69
3	53.6	25
4	14.8	3.6


*Ctrl: feed without probiotic supplementation, *B. pullicaecorum*: feed supplemented with 10^*9*^ cfu *B. pullicaecorum* per kg feed.*

## Discussion

Recently, 16S rRNA gene-based pyrosequencing analysis identified the *Ruminococcaceae* family, harboring a range of highly oxygen-sensitive butyrate-producing species, to be associated with desirable productivity outcomes since they were found highly abundant in the caecal (Stanley et al., 2016) and fecal (Singh et al., 2012) microbiota of chickens with low FCR. In our study supplementation of *B. pullicaecorum*, a member of the *Ruminococcaceae* family, resulted in a significantly lower body weight which was compensated by a significantly lower FCR. Rapid growth and heavy body weight of broilers often results in metabolic diseases such as ascites and sudden death syndrome. Also skeletal disorders resulting in immobility of the birds are often observed (Brickett et al., 2007). Therefore, slowing down body weight gain may be advantageous. This can only be of practical relevance when the improvement in feed conversion outweighs the reduced body weight in terms of economic benefit.

The subclinical form of *C. perfringens* associated- NE can adversely affects growth rate, feed conversion, and flock uniformity (Lovland and Kaldhusdal, 2001) resulting in serious economic losses on the poultry industry (Timbermont et al., 2011). In addition to the etiological agent *C. perfringens*, predisposing factors such as *Eimeria*-induced mucosal damage and diets containing high levels of proteins are required to elicit the clinical signs and lesions of NE in poultry (Fernandes da Costa et al., 2013). Those predisposing factors are hypothesized to change the gastrointestinal microbiota, providing a disrupted intestinal ecosystem in which *C. perfringens* can proliferate and cause disease (Wu et al., 2014). Pyrosequencing of 16S rRNA amplicons showed that the predominant families *Ruminococcaceae* and *Lachnospiraceae*, linked with gut health through butyrate production (Biddle et al., 2013) were the most affected bacterial phylotypes by the predisposing treatments for NE (Perez et al., 2011; Stanley et al., 2014b; Wu et al., 2014). Leeson et al. (2005) showed that birds previously fed butyrate were better in withstanding the stress of coccidial challenge (Leeson et al., 2005). Moreover butyrate was shown to induce synthesis of a subset of host defense peptides (cathelicidins) in jejunal and caecal explants from chicken origin, with immunomodulatory properties and a natural broad antimicrobial spectrum important as first line of defense (Sunkara et al., 2011). This butyrate-induced mechanism of innate host defense may account for the suppressive effect on potential pathogenic and performance-related species as observed in the present performance trial. Because butyrate-producing bacteria are depleted in the gut of chickens with NE, and butyrate has anti-inflammatory and epithelial barrier-strengthening effects (Place et al., 2005; Wang et al., 2012), administrating a strain such as *B. pullicaecorum* can restore the unbalanced microbiota composition and the butyrate production and thus counteract NE.

*B. pullicaecorum* treatment resulted in a depletion of sequences related to the genera *Enterococcus, Escherichia/Shigella, Barnesiella, Desulfovibrio*, and *Campylobacter*. All, except for *Enterococcus*, are Gram negative organisms and thus the primary source for endotoxin, also referred to as lipopolysaccharide (LPS), able to stimulate localized or systemic inflammation resulting in attenuated growth performance (Mani et al., 2012). Depending on the strain, enterococci are considered probiotic (such as *Enterococcus faecalis*; Christoffersen et al., 2012) or pathogenic organisms (such as *E. cecorum*). The latter may adversely affect performance, as it is associated with arthritis, osteomyelitis, and femoral head necrosis (Stalker et al., 2010). *Campylobacter* is considered the most common bacterial cause of human gastroenteritis and poultry has often been implicated as source (Sheppard and Maiden, 2015). Intestinal colonization of *Campylobacter* in chickens causes a negative effect on growth performance (Cisek and Binek, 2014) but, more importantly, plays an important role in carcass contamination at slaughter. In the present study, *B. pullicaecorum* supplementation was associated with a 1.47 log reduction of *Campylobacter* at slaughter age. It has been calculated that a reduction of 2 log units would reduce the public health risk by more than 90% (EFSA, 2011).

Sequences related to the genera *Lactobacillus* and *Bacteroides* were enriched in the gut of chickens treated with *B. pullicaecorum*. Lactobacilli have been shown to beneficially affect performance in broilers (Peng et al., 2016) by inducing immunomodulation and protection of the intestinal barrier via antagonistic activities against pathogens (Koenen et al., 2004; Brisbin et al., 2011). Although Gram-negative, and thus a source of endotoxin, organisms belonging to the genus *Bacteroides* are thought to play an important role in the development of the immune system, gut homeostasis and metabolism in humans (Round and Mazmanian, 2009). In infants, a low *Bacteroides* signature is associated with a decreased microbial diversity (Yassour et al., 2016) that has been associated with decreased intestinal health in general. In the chicken, as in other species, members of the *Bacteroides* genus are involved in hydrolyzing polysaccharides from plant fibers (Koropatkin et al., 2012). As such, they may contribute to the improved feed conversion.

## Conclusion

The continuous feeding of *B. pullicaecorum* suppressed the growth of undesirable microorganisms in the gut, prevented NE and improved the feed conversion in broilers. Considering the relatively small quantities of *Butyricicoccus* that were added to the feed and the relatively low abundance of the strain in the intestine, the beneficial effect of performance of *Butyricicoccus* probiotic supplementation most probably is not only due to the direct effect of its butyrate production but also due to its modulating effects on the microbiome.

## Author Contributions

FVI, RD, and FH designed the study, VE and AVP conducted the animal trials. VE performed the qPCR analysis and JW, MJ, GF, and JR carried out bioinformatics analysis of the data. VE drafted the manuscript. All authors edited the manuscript and approved the final version of the manuscript.

## Conflict of Interest Statement

The authors declare that the research was conducted in the absence of any commercial or financial relationships that could be construed as a potential conflict of interest.

The reviewer LB and handling Editor declared their shared affiliation, and the handling Editor states that the process nevertheless met the standards of a fair and objective review.

## Footnotes

1 http://hannonlab.cshl.edu/fastx_toolkit/.
